# Deciphering influenza B virus-derived peptides and their presentation by HLA class I molecules

**DOI:** 10.1042/BST20260075

**Published:** 2026-07-31

**Authors:** Janesha C. Maddumage, Georgia R. Dow, Lawton D. Murdolo, Samuel Liwei Leong, Demetra S.M. Chatzileontiadou, Emma J. Grant, Stephanie Gras

**Affiliations:** 1 Infection and Immunity Program, La Trobe Institute for Molecular Science (LIMS), La Trobe University, Bundoora, Victoria 3086, Australia; 2 Department of Biochemistry and Chemistry, School of Agriculture, Biomedicine and Environment (SABE), La Trobe University, Bundoora, Victoria 3086, Australia; 3 Department of Biochemistry and Molecular Biology, Biomedicine Discovery Institute, Monash University, Clayton, Victoria 3800, Australia

**Keywords:** epitope presentation, FLUBV, HLA, Influenza, structural biology, T-cells

## Abstract

Despite influenza vaccines being widely available, influenza still causes significant morbidity and mortality annually. Vaccines typically induce humoral-mediated protection against rapidly mutating surface glycoproteins, necessitating that they be updated and administered each year. In contrast, CD8^+^ T cells, which can control and clear viral infections, can recognise more conserved viral epitopes. Therefore, there is considerable interest in understanding CD8^+^ T cell responses to influenza virus for the development of future vaccines and therapeutics. Although *Alphainfluenzavirus influenzae* (FLUAV) and *Betainfluenzavirus influenzae* (FLUBV) co-circulate in humans and both contribute to seasonal epidemics, there is limited data regarding CD8^+^ T cell responses to FLUBV. This knowledge gap spans both immunological and molecular insights. In the present review, we summarise the current knowledge of FLUBV-derived CD8^+^ T cell epitopes at both cellular and molecular levels, in comparison with FLUAV. Collectively, this highlights the limited data available on FLUBV, despite its significant role in human influenza infections.

## Introduction

Influenza is a respiratory disease caused by viruses from the Orthomyxoviridae family. These enveloped, single-stranded RNA viruses include *Alphainfluenzavirus influenzae* (FLUAV) and *Betainfluenzavirus influenzae* (FLUBV), circulate seasonally in humans and are predominantly the causative agents of seasonal ‘flu’ [1,2]. *Gammainfluenzavirus influenzae* also circulate in humans, although severe disease is generally limited to children [3], while *Deltainfluenzavirus influenzae* circulates among cattle and swine and periodically spills over into other animal species [4]. FLUAV and FLUBV both circulate concurrently in humans and together contribute to annual epidemics, causing significant global morbidity and mortality in humans, with up to 650,000 deaths estimated annually [5,6]. They also have a high impact on the global economy, costing over 11 billion USD each year [7]. Further, FLUAV is well recognised for its pandemic potential, as evidenced by major outbreaks such as the 1918 H1N1 (Spanish Flu) and the 2009 H1N1 (Swine Flu) pandemics [8,9]. FLUAV strains are divided into subtypes based on which haemagglutinin (HA) or neuraminidase (NA) they carry [2]. In addition to infecting humans, FLUAV also circulates in wild animal populations. The diversity in FLUAV therefore comes from both antigenic shift, where novel HA or NA antigens are acquired during gene segment recombination between distinct viruses [10,11], and the accumulation of single nucleotide polymorphisms in these antigens known as antigenic drift [2]. Conversely, FLUBV predominantly infects humans and has no established animal reservoir [12], and as a result, FLUBV diversity is due only to antigenic drift with FLUBV strains divided into lineages, B/Victoria-like and B/Yamagata-like. Interestingly, detection of B/Yamagata viruses dropped dramatically from 2020 during the COVID-19 pandemic, and detection of these viruses has not been reported in World Health Organisation (WHO) global surveillance data since 2023 [13]. In fact, several reports suggest this lineage may have been eradicated, although ongoing surveillance, particularly in low-income countries with higher FLUBV incidence and less robust surveillance systems, is still needed to confirm this [13,14]. While the possibility of eradicating one of the FLUBV lineages is promising, B/Victoria lineage viruses have continued to circulate globally, contributing to global health and economic burden. A major concern with FLUBV infections is how they disproportionately impact children, with studies reporting a greater than 2-fold higher incidence of FLUBV in children than other age groups, as well as increased rates of complications, hospitalisation and death in children [15–18].

Since both FLUAV and FLUBV contribute to influenza disease burden [5,15,19], both are present in seasonal influenza vaccines, which are modified annually based on the dominant FLUAV and FLUBV variants circulating in the opposite hemisphere in the previous winter [20,21]. Traditionally, these vaccines have been quadrivalent formulations comprising two FLUAV strains (H1N1 and H3N2) and two FLUBV strains (Yamagata and Victoria lineages). However, due to the recent decline in FLUBV Yamagata detection, the World Health Organisation no longer recommends its inclusion in vaccines [22]. These influenza vaccines primarily elicit a strong and neutralising antibody response produced by B cells [23] and display variable effectiveness annually, varying from 2% to 85% vaccine effectiveness (VE) depending on the assessed virus strain [24]. There is great interest in the development of novel influenza vaccines that provide longer-lasting protection against multiple influenza virus strains. During an influenza infection, both CD4^+^ and CD8^+^ T cells are activated, providing protection and long-term immune memory [25–28]. As such, it is important to understand the T cell response towards both FLUAV and FLUBV to improve future vaccine design or therapeutics to limit the health burden of influenza disease.

CD8^+^ T cells are known as cytotoxic or ‘killer’ T cells due to their ability to directly kill infected host cells [29–31]. They also produce a variety of cytokines, such as interferon-γ (IFNγ), tumour necrosis factor (TNF), and interleukin-2 (IL-2), which characterise the effector response [31]. Robust CD8^+^ T cell responses, including polyfunctional CD8^+^ T cells (production of more than one of IL-2, TNF, and IFNγ) [32], and CD8^+^ T cells with diverse T cell receptor (TCR) repertoires [33], have been implicated in the reduction of disease severity and viral clearance in a variety of viral infections [32,34]. In particular, the role and importance of CD8^+^ T cells in the context of influenza virus infection have been explored in several murine and human studies [29,35–38].

CD8^+^ T cells recognise viral peptides via their TCR, presented by major histocompatibility complex (MHC) class I molecules, also named human leukocyte antigen class I (HLA-I) in humans [39]. HLA-I molecules are expressed on all nucleated cells, and there are three classical HLA-I molecules: HLA-A, HLA-B, and HLA-C [40,41]. HLA-I molecules are the most polymorphic molecule in humans and are central to antiviral immunity. Structurally, HLA-I molecules consist of a membrane-bound heavy chain (α-chain) composed of three extracellular domains (α1, α2, and α3) associated non-covalently with the β2-microglobulin [41]. The α1- and α2-domains of HLA-I together comprise the antigen-binding cleft or groove in which the viral peptide sits to be presented to CD8^+^ T cells. The antigen-binding cleft is formed by two α-helices that form the ‘wall’ of the cleft and β-strand sheets at the bottom of the groove that form the ‘floor’. Each HLA-I groove is composed of peptide binding pockets (A–F) [42], with two primary anchor pockets, B and F. These accommodate the peptide primary anchor residues at position 2 (P2) and the last position (PΩ), respectively, and are key for peptide stability. The HLA-I binding cleft is closed at both N and C termini, limiting the length of the bound peptide to an optimal 8–10 residues. However, longer peptides can be presented [43]. Overall, HLA-I polymorphism, peptide stability, and peptide anchor residue compositions are major determinants, dictating the breadth of peptides that a specific HLA-I molecule will present [42].

As peptide presentation by HLA-I molecules is the first step to activating CD8^+^ T cells, understanding the breadth of viral peptides presented by HLA molecules and the interplay between the two can help better define the immune response towards viral infection. While FLUAV-derived peptides and their resulting CD8^+^ T cell responses have been reasonably well studied, there is significantly less known about FLUBV-derived peptides and CD8^+^ T cells, and this is the focus of the present review.

## FLUBV-derived peptides and epitopes presented by HLA-I molecules

To begin exploring FLUBV-derived peptides and their presentation by HLA-I molecules, we first collated the FLUBV-derived peptides published on the Immune Epitope Database (IEDB) [44]. We collated all known FLUBV-derived peptides (known hereafter as ‘peptides’) reported to have either positive HLA-ligand (HLA-I binding) assays and/or those that were reported to induce a functional T cell response (immunogenic, this subset is referred to hereafter as ‘epitopes’) (Table 1). We also collated the FLUAV-derived peptides for comparison (Table 2). Peptide length, peptide origin, and immunogenicity were also recorded. To narrow the focus to naturally occurring FLUBV- and FLUAV-derived peptides, those with modified residues were excluded (e.g., oxidation, acetylation, and deamidation). To restrict the analysis to peptides likely to be restricted to HLA-I molecules, only 8–12-mer peptides were included. If a shorter minimal epitope was overlapping with a longer 11–12-mer peptide, the longer of the peptides was also excluded.

**Table 1:** FLUBV peptide sequence, length, and source protein

FLUBV peptides	Peptide length (aa)	FLUBV protein source	HLA-I restriction
EAINKITK	8	HA	
ILHEVRPV	8	HA	
PLTTTPTK	8	HA	
VTNGNGFF	8	HA	
WVKTPLKL	8	HA	
AINKITKNL	9	HA	
GLDNHTILL	9	HA	HLA-A*02:01
GMIAGWHGY	9	HA	
GRIVVDYMV	9	HA	
GVAVAADLK	9	HA	
HYVSQIGGF	9	HA	
IPLTTTPTK	9	HA	
IPSARVSIL	9	HA	
ITSSNSPHV	9	HA	
IWVKTPLKL	9	HA	
KVDDLRADT	9	HA	
LPQSGRIVV	9	HA	
NYLSKRYFI	9	HA	HLA-A*24:02
SILHEVRPV	9	HA	
SLTIEVPYI	9	HA	
TFDAGEFSL	9	HA	
VYMVSRDNV	9	HA	
YGDSKPQKF	9	HA	
YTSHGAHGV	9	HA	
YYSTAASSL	9	HA	HLA-A*24:02
AVLLSNEGII	10	HA	
FPIMHDRTKI	10	HA	
IINSEDEHLL	10	HA	
KMHQEDPTKL	10	HA	
LYGDSKPQKF	10	HA	HLA-A*24:02
PYYTGEHAKA	10	HA	
TAASSLAVTL	10	HA	
VIPLTTTPTK	10	HA	
LLSYQLSISW	10	Matrix	
FGDTIAYL	8	Matrix protein 1 (M1)	
KMRRCVSF	8	M1	
AARSSVPGV	9	M1	
ALIGASICF	9	M1	HLA-B*15:01
SALEWIKNK	9	M1	HLA-A*11:01
VSFHEAFEI	9	M1	
YLNPGNYSM	9	M1	HLA-A*02:01
ALIGASICFL	10	M1	NA
IQKALIGASI	10	M1	NA
KALIGASICF	10	M1	HLA-B*15:01
MYLNPGNYSM	10	M1	HLA-A*24:02
SSMGNSALVK	10	M1	
TIAYLLSLTE	10	M1	
SILRHSYQK	9	Matrix protein 2 (M2)	
TIGHLNQIK	9	M2	
KLGKIPTV	8	Neuraminidase (NA)	
LYSDILLK	8	Neuraminidase (NA)	
AYTDTYHSY	9	Neuraminidase (NA)	
GVSECRFLK	9	Neuraminidase (NA)	
HSYANNILR	9	Neuraminidase (NA)	
LLYSDILLK	9	Neuraminidase (NA)	
LYSDILLKF	9	Neuraminidase (NA)	
STIQTLTLF	9	Neuraminidase (NA)	
TVTGVNMAL	9	Neuraminidase (NA)	
TYHSYANNI	9	Neuraminidase (NA)	HLA-A*24:02
ALLKIKYGEA	10	Neuraminidase (NA)	
PFVPILFEQL	10	Neuraminidase (NA)	
SMEEPGWYSF	10	Neuraminidase (NA)	
AYDQSGRL	8	Non-structural protein 1 (NS1)	
AYDQSGRLV	9	NS1	
ILNSLFERL	9	NS1	
KSEPEGTRM	9	NS1	
NVLSLRVLV	9	NS1	
SQFGQEHRL	9	NS1	HLA-A*02:01
ALDYPGQDRL	10	NS1	
RVLVNGTFLK	10	NS1	HLA-A*11:01
IQSQFEQLK	9	Nuclear export protein (NEP)	
RIDDNILFH	9	NEP	
RLVTEELYL	9	NEP	
VLMKDIQSQF	10	NEP	
FLKEEVKT	8	Nucleoprotein (NP)	
IYAKIPQL	8	NP	
KLGEFYNQ	8	NP	
LGEFYNQM	8	NP	
AAYEDLRVL	9	NP	
ADRGLLRDI	9	NP	NA
ALKCKGFHV	9	NP	NA
ATGVAIKGG	9	NP	
ATLAPPSNK	9	NP	
CFQRSKALK	9	NP	
FAVERPIAL	9	NP	
KLGEFYNQM	9	NP	HLA-A*02:01
NLIQNAHAV	9	NP	
RPIIRPATL	9	NP	NA
SKVVLPISI	9	NP	
SPIRVTFLK	9	NP	
TLLARSMVV	9	NP	
VTFLKEEVK	9	NP	
VVRPSVASK	9	NP	HLA-A*03:01
YEDLRVLSA	9	NP	NA
YFSPIRITF	9	NP	NA
YFSPIRVTF	9	NP	HLA-A*24:02; HLA-A*11:01
IYAKIPQLGF	10	NP	
IYFSPIRITF	10	NP	NA
IYFSPIRVTF	10	NP	HLA-A*24:02
KLGEFYNQMM	10	NP	HLA-A*02:01
KTNGNAFIGK	10	NP	HLA-A*11:01
QTIPNFFFGR	10	NP	
SAPQQKALVD	10	NP	
STFAGSTLPR	10	NP	
VLSALTGTEF	10	NP	
VVLPISIYAK	10	NP	
VVVRPSVASK	10	NP	
FQTTIIQKA	9	Polymerase acidic protein (PA)	
GTQEGKLVK	9	PA	
KYVLFHTSL	9	PA	HLA-A*24:02
SLFVSGREK	9	PA	
TVMMKYVLF	9	PA	
ATGDGLTYQK	10	PA	
FLDEEGKAYT	10	PA	
IGTQEGKLVK	10	PA	
KNDEEILI	8	Polymerase basic protein 2 (PB2)	
KTDQFIKL	8	PB2	
GQYSGFARA	9	PB2	
ITFGPVERV	9	PB2	
IYHPGGNKL	9	PB2	
MYQLQRYFL	9	PB2	HLA-A*24:02
NPLELAVEI	9	PB2	
RVLLNPLTK	9	PB2	
RVYESFFLR	9	PB2	
SGFARAVLK	9	PB2	
TYQWVLKNL	9	PB2	
TYQWVMKNL	9	PB2	NA
VFSQDTRMF	9	PB2	
RVYESFFLRK	10	PB2	
YFLNRSNDLF	10	PB2	HLA-A*24:02
FNMLSTVLG	9	RNA-directed RNA polymerase catalytic subunit (PB1)	NA
KVKDKITKV	9	PB1	
LSPGMMMGM	9	PB1	
NFAMELPSF	9	PB1	HLA-A*24:02
PEMTFFSVK	9	PB1	
VADGGPNIY	9	PB1	NA
YRDGFVSNF	9	PB1	
YSHGTGTGY	9	PB1	
FYRDGFVSNF	10	PB1	HLA-A*24:02
GVAALGIKNI	10	PB1	
KYNLMDPEYK	10	PB1	
GAMDELHNEIL	11	HA	
GFLEGGWEGMI	11	HA	
SLNDDGLDNHT	11	HA	
KNLNSLSELEVK	12	HA	
SGCFPIMHDRTK	12	HA	
GVLRSLGASQK	11	M1	
IIEGLSAEEIIK	12	M2	
GVDGPDNNALLK	12	NA	
GTFLKHPNGYK	11	NS1	
LFMDPSAGIEGF	12	NS1	
ATDDKKTEFQK	11	NP	
GQISCSPVFAV	11	NP	
GTIDKAPEEIT	11	NP	
KEGKEEIDHNK	11	NP	
MYKTTMGSDGF	11	NP	
VGLDPSLISTF	11	NP	
VYNMVVKLGEF	11	NP	
MIFSYNQDYSL	11	PA	
SLDEEGKGRVL	11	PA	
YLADLFDYKTK	11	PA	
GIWDGEEEFHV	11	PB2	
TTVDQYNIIRK	11	PB2	
TYGPIGDTEGF	11	PB2	HLA-A*24:02

FLUBV-derived peptides published on the IEDB [44] as having positive ‘T cell’ (immunogenic) or ‘MHC-ligand’ (HLA-I binding) assays were collated. Peptides with modified residues were excluded. Peptide length (number of amino acids [aa]) and the protein each peptide was derived from was also recorded. Immunogenic sequences are shown with purple shading. HLA-I restriction is provided for immunogenic epitopes where available, with ‘NA’ indicating that the HLA-I information is not available.

**Table 2:** FLUAV peptide sequence, length, source protein, and sequence identity to recent FLUBV vaccine strains

FLUAV peptides	Peptide length (aa)	FLUAV source protein	Sequence identity to FLUBV (Min%–Max %)
DNFDKLYI	8	HA	
FYKNLIWL	8	HA	
ITDDQIEV	8	HA	
KLRGVAPL	8	HA	
KSGYKDWI	8	HA	
NVTVTHSV	8	HA	
SFERFEIF	8	HA	
SFYKNLIW	8	HA	
VPRYAFAM	8	HA	
YASLRSLV	8	HA	
YPYDVPDY	8	HA	
AEDMGNGCF	9	HA	
AVPNGTLVK	9	HA	
CYPGYFADY	9	HA	
CYPYDVPDY	9	HA	
DEFLKVPEW	9	HA	
DTVDTVLEK	9	HA	
FAISCFLLC	9	HA	
FHDSNVKNL	9	HA	
FLDIWTYNA	9	HA	
FQNVNKITY	9	HA	
FQNVNRITY	9	HA	
GIAPLQLGK	9	HA	33.33%–33.33%
GIFGAIAGF	9	HA	
GIHHPSNSK	9	HA	33.33%–33.33%
GKLNRLIGK	9	HA	
GMMDGWYGF	9	HA	
GRINYYWTL	9	HA	
GRIQDLEKY	9	HA	
GRITVSTKR	9	HA	
GRVTVSTKR	9	HA	
GTFSLNAAK	9	HA	
GTGQAADLK	9	HA	
HAGAKSFYK	9	HA	
HANNSTDTV	9	HA	
HHAVPNGTL	9	HA	
IAGFIEGGW	9	HA	
IAGFIENGW	9	HA	
IAPWYAFAL	9	HA	
IEVTNATEL	9	HA	
ILDGENCTL	9	HA	
ILDGKNCTL	9	HA	
IPSIQSRGL	9	HA	
ITYGACPRY	9	HA	
IVFMWAIHH	9	HA	
KEFSEVEGR	9	HA	
KFHQIEKEF	9	HA	
KLRMVTGLR	9	HA	
KMNIQFTAV	9	HA	
KMNTQFEAV	9	HA	
KSFFSRLNW	9	HA	
LEDSHNGKL	9	HA	
LEKTHNGKL	9	HA	
LENERTLDF	9	HA	44.44%–44.44%
LIAPRGYFK	9	HA	
LLLAIVSLV	9	HA	
LPFHNVHPL	9	HA	
LPFQNIHPI	9	HA	
LPFQNVHPV	9	HA	
LRNIPSIQS	9	HA	
LSTASSWSY	9	HA	
LVGGREWSY	9	HA	
LVSLGAISF	9	HA	
MTIIFLILM	9	HA	44.44%–44.44%
NIQFTAVGK	9	HA	
NTLKLATGM	9	HA	
PVTIGECPK	9	HA	
QILSIYSTV	9	HA	
QLKYKYPAL	9	HA	
RDEALNNRF	9	HA	
REKIDGVKL	9	HA	
RLIEKTNEK	9	HA	
RLIGKTNEK	9	HA	
RLNWLTHLK	9	HA	
RSKAFSNCY	9	HA	
RSKAYSNCY	9	HA	
RVIEKTNEK	9	HA	
SMGVYQILA	9	HA	
SSVSSFERF	9	HA	
TIGECPKYV	9	HA	
TIVKPGDVL	9	HA	
TLCLGHHAV	9	HA	
TSADQQSLY	9	HA	
TVLEKNVTV	9	HA	
TYPVLNVTM	9	HA	44.44%–44.44%
VALENQHTI	9	HA	
VLLVSLGAI	9	HA	55.56%–55.56%
VLMENERTL	9	HA	
VSLGAISFW	9	HA	
WTGMVDGWY	9	HA	
YNAELLVAL	9	HA	
YNAELLVLL	9	HA	
YPKLKNSYV	9	HA	
YVFVGTSRY	9	HA	
YVKQNTLKL	9	HA	
YVSVVSSHY	9	HA	
CPKYVRSAKL	10	HA	
DQHGRMNYYW	10	HA	
FLNVPEWSYI	10	HA	
GLFGAIAGFI	10	HA	80.00%–80.00%
ISFAISCFLL	10	HA	
KLESMGIYQI	10	HA	
KLYQNPTTYI	10	HA	40.00%–40.00%
LPVRSWSYIV	10	HA	
LRNIPSIQSR	10	HA	
LRNVPQIESR	10	HA	
LTDSEMNKLF	10	HA	
LTEKEGSYPK	10	HA	
MRNVPEKQTR	10	HA	
NSNGNLIAPR	10	HA	
NVKNLYEKVK	10	HA	
RLYQNPTTYI	10	HA	30.00%–30.00%
RMNYYWTLLK	10	HA	
RYSKKFKPEI	10	HA	
SLPFQNIHPV	10	HA	
STSADQQSLY	10	HA	
TPLGAINSSL	10	HA	
VTAACSHAGK	10	HA	50.00%–50.00%
WILWISFAIS	10	HA	
WISFAISCFL	10	HA	
WSYNAELLVA	10	HA	
YQNPTTYISV	10	HA	
YRDEALNNRF	10	HA	
AVGKEFNKLEK	11	HA	
ITNKVNSVIEK	11	HA	
IYSTVASSLVL	11	HA	
LPYQNIHPVTI	11	HA	27.27%–27.27%
LVASSGTLEFI	11	HA	
RTLDFHDSNVK	11	HA	27.27%–27.27%
SVRNGTYDYPK	11	HA	
ATELVQSSSTGK	12	HA	
PRYVRQNTLRLA	12	HA	
STQAAIDQINGK	12	HA	
AAEAMEVA	8	M1	
ALASCMGL	8	M1	
ASCMGLIY	8	M1	37.50%–37.50%
GILGFVFT	8	M1	
GILGFVTL	8	M1	
ILGFVFTL	8	M1	37.50%–37.50%
LTEVETYV	8	M1	
NDLLENLQ	8	M1	
NTDLEVLM	8	M1	
TEVETYVL	8	M1	25.00%–25.00%
ALASCMGLI	9	M1	33.33%–33.33%
ALSYSAGAL	9	M1	33.33%–33.33%
DLLENLQAY	9	M1	
DLLENLQTY	9	M1	
FHGAKEIAL	9	M1	
FVFTLTVPS	9	M1	
FYGAKEIAL	9	M1	
GAKEIALSY	9	M1	
GAKEISLSY	9	M1	
GALASCMGL	9	M1	
GILEFVFTL	9	M1	44.44%–44.44%
GILGFIFTL	9	M1	33.33%–33.33%
GILGFVFLT	9	M1	33.33%–33.33%
GILGFVFTL	9	M1	33.33%–33.33%
GILGFVYTL	9	M1	33.33%–33.33%
GILGLVFTL	9	M1	33.33%–33.33%
GILGVFVTL	9	M1	
GILGVVFTL	9	M1	33.33%–33.33%
GIWGFVFTL	9	M1	33.33%–33.33%
GLIYNRMGA	9	M1	33.33%–33.33%
GMLGFVFTL	9	M1	33.33%–33.33%
GTLGFVFTL	9	M1	33.33%–33.33%
GVLGFVFTL	9	M1	33.33%–33.33%
ILGFVFTLT	9	M1	33.33%–33.33%
ILSPLTKGI	9	M1	33.33%–33.33%
IRHENRMVL	9	M1	44.44%–44.44%
ITFHGAKEI	9	M1	
KRMGVQMQR	9	M1	
KRMGVQVQR	9	M1	
LGFVFTLTV	9	M1	33.33%–33.33%
LLTEVETYV	9	M1	33.33%–33.33%
LTKGILGFV	9	M1	33.33%–33.33%
LYKKLKREI	9	M1	
MVLASTTAK	9	M1	
NMDRAVKLY	9	M1	
QARQMVQAM	9	M1	
QARRMVQAM	9	M1	
QMVTTTNPL	9	M1	
REITFHGAK	9	M1	
RIRFVQNAL	9	M1	
RLEDVFAGK	9	M1	44.44%–44.44%
RMGAVTTEV	9	M1	
RMGVQMQRF	9	M1	
RMVLASTTA	9	M1	55.56%–55.56%
RRRFVQNAL	9	M1	
SCMGLIYNR	9	M1	33.33%–33.33%
SIIPSGPLK	9	M1	33.33%–44.44%
SSSTEVETY	9	M1	
TTAKAMEQM	9	M1	
VETYVLSII	9	M1	
VETYVLSIV	9	M1	
VLASTTAKA	9	M1	55.56%–55.56%
VTTEVAFGL	9	M1	33.33%–33.33%
WILGFVFTL	9	M1	44.44%–44.44%
YSAGALASC	9	M1	
YSGPLKAEI	9	M1	
ALASCMGLIY	10	M1	40.00%–40.00%
ASCMGLIYNR	10	M1	30.00%–30.00%
AYQKRMGVQM	10	M1	30.00%–30.00%
EAMEVASQAR	10	M1	40.00%–40.00%
GILGFVFTLT	10	M1	30.00%–30.00%
GTGIQITWTK	10	M1	
ILGFVFTLTV	10	M1	30.00%–30.00%
ILSPLTKGIL	10	M1	40.00%–40.00%
ITFHGAKEIA	10	M1	
KNDLLENLQA	10	M1	
KTRPILSPLT	10	M1	
LASCMGLIYN	10	M1	40.00%–40.00%
LKNDLLENLQ	10	M1	
LLENLQAYQK	10	M1	
LLTEVETYVL	10	M1	
LSIIPSGPLK	10	M1	
PILSPLTKGI	10	M1	
QMVTTTNPLI	10	M1	
RMGVQMQRFK	10	M1	
RMVLASTTAK	10	M1	60.00%–60.00%
RQMATTTNPL	10	M1	
SEQAAEAMEV	10	M1	40.00%–40.00%
SLLTEVETYV	10	M1	
SLQGRTPILR	10	M1	
TEVETYVLSI	10	M1	30.00%–30.00%
TFHGAKEVSL	10	M1	40.00%–40.00%
ALMEWLKTRPI	11	M1	
GLVCATCEQIA	11	M1	
GTHPSSSAGLK	11	M1	
LYKKLKREMTF	11	M1	36.36%–36.36%
LYRKLKREITF	11	M1	36.36%–36.36%
SYSTGALASCM	11	M1	
TTNPLIRHENR	11	M1	
VLMEWLKTRPI	11	M1	
ITDRLFFK	8	M2	
FFKCIYRLF	9	M2	
GILHLILWI	9	M2	33.33%–33.33%
IGILHLILW	9	M2	
IIGILHLIL	9	M2	44.44%–44.44%
ILDRLFFKC	9	M2	44.44%–44.44%
KSMREEYRK	9	M2	33.33%–33.33%
LILWILDRL	9	M2	33.33%–33.33%
LLTEVETPI	9	M2	22.22%–33.33%
RLFFKCIYR	9	M2	
RLFKHGLKR	9	M2	
SIIGILHLI	9	M2	
VETPIRNEW	9	M2	33.33%–33.33%
WILDRLFFK	9	M2	44.44%–44.44%
YRKEQQNAV	9	M2	
YRLFKHGLK	9	M2	
GGPSTEGVPK	10	M2	
GILHLILWIL	10	M2	
RLFFKCIYRR	10	M2	20.00%–20.00%
CIYRRFKYFLK	11	M2	
CIYRRFKYGLK	11	M2	
GYICSGIF	8	NA	
APSPYNSRF	9	NA	
CVNGSCFTV	9	NA	33.33%–33.33%
DTVHDRTPY	9	NA	
DWSGYSGSF	9	NA	
EMIWDPNGW	9	NA	
ETFKVIGGW	9	NA	
FLMQIAILV	9	NA	
GADINLMPI	9	NA	
GLISLILQI	9	NA	
GMGWLTIGI	9	NA	
GRADTKILF	9	NA	
GSNRPWVSF	9	NA	
GVKGFSFKY	9	NA	
GWTETDSSF	9	NA	
HLECRTFFL	9	NA	
ISEKLRSGY	9	NA	
LMNELGVPF	9	NA	
NELGVPFHL	9	NA	
NSDTVDWSW	9	NA	
QIAILVTTV	9	NA	33.33%–33.33%
RLVDSIGSW	9	NA	
RLVDSIVSW	9	NA	
RPCFWVELI	9	NA	
RTFFLTQGA	9	NA	
SCINRCFYV	9	NA	
TIHDRIPHR	9	NA	
TLHFKQYEF	9	NA	
TLLMNELGV	9	NA	
VMTDGPANK	9	NA	
VQHPELTGL	9	NA	
VSFDQNLDY	9	NA	
VSFNQNLEY	9	NA	
VTTVTLHFK	9	NA	
YPRYPGVRC	9	NA	
YRTLLMNEL	9	NA	
ASYKIFRIEK	10	NA	
GPDNGAVAVL	10	NA	
IVSSYVCSGL	10	NA	
KYNGIITDTI	10	NA	
MQIAILVTTV	10	NA	
RPWVSFDQNL	10	NA	
RYGNGVWIGR	10	NA	40.00%–40.00%
SLCPIRGWAI	10	NA	30.00%–30.00%
SPYNSRFESV	10	NA	
SWPDGAELPF	10	NA	40.00%–40.00%
WVSFNQNLEY	10	NA	
SPYRTLMSCPI	11	NA	
FTEEGAIV	8	NS1	
IMDKNIIL	8	NS1	
MLMPRQKI	8	NS1	
VLKANFSV	8	NS1	
AIMDKNIIL	9	NS1	33.33%–33.33%
AIMDKNIML	9	NS1	33.33%–33.33%
AIMDKNITL	9	NS1	33.33%–33.33%
AIMDKTIIL	9	NS1	33.33%–33.33%
AIMDKVIIL	9	NS1	33.33%–33.33%
AIMEKNIIL	9	NS1	33.33%–33.33%
AIMEKNIML	9	NS1	
AIMEKNIVL	9	NS1	33.33%–33.33%
AIVDKNITL	9	NS1	33.33%–33.33%
ALKMTMASV	9	NS1	
AVMEKNIVL	9	NS1	33.33%–33.33%
ETIVLLRAF	9	NS1	
FLWHVRKRV	9	NS1	
GEISPLPSL	9	NS1	33.33%–33.33%
GLEWNDNTV	9	NS1	
GVLIGGLEW	9	NS1	
IASVPTSRY	9	NS1	
IFDRLETLI	9	NS1	
IILKANFSV	9	NS1	
IMLKANFSV	9	NS1	
KQIVERILK	9	NS1	
KRKMARTAR	9	NS1	
LPSFPGHTI	9	NS1	
LPSLPGHTA	9	NS1	
MASTPASRY	9	NS1	
MSRDWFMLM	9	NS1	
QELSDAPFL	9	NS1	
RLRRDQRSL	9	NS1	
SETLQRFAW	9	NS1	33.33%–33.33%
TMASVPASR	9	NS1	
AIMEKNIMLK	10	NS1	
AIMEKNIVLK	10	NS1	
ENGRPPLTPK	10	NS1	
FQVDCFLWHV	10	NS1	30.00%–30.00%
ILKEESDEAL	10	NS1	
IMDKNIILKA	10	NS1	30.00%–30.00%
IPKQKVAGPL	10	NS1	
IVDKNITLKA	10	NS1	30.00%–30.00%
MPRQKIIGPL	10	NS1	
MTIASVPTSR	10	NS1	
VPASRYLTDM	10	NS1	
ALQLLLEV	8	Nonstructural protein 2 (NS2)	37.50%–37.50%
QEIRTFSF	8	Nonstructural protein 2 (NS2)	25.00%–25.00%
LLFEVEQEI	9	Nonstructural protein 2 (NS2)	
MVTRFESLK	9	Nonstructural protein 2 (NS2)	
NSFEQITFM	9	Nonstructural protein 2 (NS2)	
RLKTTENSF	9	Nonstructural protein 2 (NS2)	
SSFQDILLR	9	Nonstructural protein 2 (NS2)	
TQFESLKIY	9	Nonstructural protein 2 (NS2)	
VSSFQDILL	9	Nonstructural protein 2 (NS2)	
ITFMQALQLL	10	Nonstructural protein 2 (NS2)	30.00%–30.00%
QEIRTFSFQL	10	Nonstructural protein 2 (NS2)	30.00%–30.00%
NTVSSFQV	8	NEP	
TQFESLKL	8	NEP	
VEQEIRTF	8	NEP	25.00%–37.50%
DSLGEAVMR	9	NEP	
FMQALHLLL	9	NEP	
FMQALQLLL	9	NEP	
FQVDCFLWH	9	NEP	
LLLEVEQEI	9	NEP	
MRMGDLHLL	9	NEP	
TFMQALHLL	9	NEP	33.33%–33.33%
TVSSFQDIL	9	NEP	
VMRMGDLHY	9	NEP	
MQALQLLLEV	10	NEP	
QAIMDKNIIL	10	NEP	
TENSFEQITF	10	NEP	30.00%–30.00%
FEDLRVLS	8	NP	87.50%–87.50%
LPFERATV	8	NP	
LRSRYWAI	8	NP	37.50%–37.50%
RYWAIRTR	8	NP	37.50%–50.00%
TELKLSDY	8	NP	
YERMCNIL	8	NP	37.50%–37.50%
AAFEDLRLL	9	NP	
AAFEDLRVL	9	NP	88.89%–88.89%
AAGAAVKGV	9	NP	
AEIEDLIFL	9	NP	66.67%–66.67%
AEIEDLIFS	9	NP	55.56%–55.56%
ALRSRYWAI	9	NP	
AMDSNTLEL	9	NP	33.33%–33.33%
ATFSKGNFK	9	NP	
ATYQRTRAL	9	NP	
AVKGVGTMV	9	NP	
CLPACVYGL	9	NP	33.33%–33.33%
CTELKLNDY	9	NP	44.44%–44.44%
CTELKLSDH	9	NP	
CTELKLSDN	9	NP	
CTELKLSDY	9	NP	55.56%–55.56%
CTELKLTDQ	9	NP	33.33%–33.33%
CTELKLTDY	9	NP	44.44%–44.44%
DATAGLTHI	9	NP	55.56%–55.56%
DMRTEIIRM	9	NP	
DPKKTGGPI	9	NP	
DRYWAIRTR	9	NP	33.33%–44.44%
EDLTFLARS	9	NP	88.89%–88.89%
ELKSRYWAI	9	NP	33.33%–33.33%
ELRSRYWAI	9	NP	33.33%–33.33%
FEDLRLLSF	9	NP	66.67%–66.67%
FEDLRVLSF	9	NP	77.78%–77.78%
FEDLRVSSF	9	NP	66.67%–66.67%
FEKEGYSLV	9	NP	
FLARSALIL	9	NP	55.56%–55.56%
FQGRGVFEL	9	NP	33.33%–44.44%
FRGRGVFEL	9	NP	
FSVQRNLPF	9	NP	
FYIQMCTEL	9	NP	44.44%–44.44%
GERQNATEI	9	NP	44.44%–44.44%
GINDRNFWR	9	NP	
GMDPRMCSL	9	NP	44.44%–44.44%
GQISIQPTF	9	NP	
GRWMRELVL	9	NP	
GTKVVPRGK	9	NP	
GVFELSDEK	9	NP	
GYDFEREGY	9	NP	
HSNLNDATY	9	NP	44.44%–44.44%
HSNLNDTTY	9	NP	
ILKGKFQTA	9	NP	44.44%–44.44%
ILRGSIAHK	9	NP	44.44%–44.44%
ILRGSVAHK	9	NP	55.56%–55.56%
KLSDYEGRL	9	NP	33.33%–33.33%
KLSTRGVQI	9	NP	33.33%–33.33%
KMIDGIGRF	9	NP	
KSCLPACVY	9	NP	44.44%–44.44%
KTFFPIYKR	9	NP	44.44%–44.44%
KTGGPIYKR	9	NP	66.67%–66.67%
KTGGPIYRR	9	NP	55.56%–55.56%
KWMRELVLY	9	NP	33.33%–33.33%
LELRSRYWA	9	NP	33.33%–33.33%
LIFLARSAL	9	NP	55.56%–55.56%
LLQNSQVYS	9	NP	
LMQGSTLPR	9	NP	
LPFDIATIM	9	NP	33.33%–33.33%
LPFDKATIM	9	NP	33.33%–33.33%
LPFDKITIM	9	NP	
LPFDKPTIM	9	NP	33.33%–44.44%
LPFDKQTIM	9	NP	
LPFDKSTIM	9	NP	33.33%–33.33%
LPFDKSTVM	9	NP	44.44%–44.44%
LPFDKTTIM	9	NP	33.33%–33.33%
LPFDKTTVM	9	NP	
LPFDRPTIM	9	NP	33.33%–44.44%
LPFDRTTIM	9	NP	33.33%–33.33%
LPFDRTTVM	9	NP	
LPFEKSIVM	9	NP	
LPFEKSTIM	9	NP	33.33%–33.33%
LPFEKSTVM	9	NP	44.44%–44.44%
LPFEKTTIM	9	NP	
LPFERATII	9	NP	
LPFERATIM	9	NP	44.44%–44.44%
LPFERATVL	9	NP	44.44%–44.44%
LPFERATVM	9	NP	44.44%–44.44%
LPFERSTIM	9	NP	33.33%–33.33%
LPFERSTVM	9	NP	
LPFGKTTIM	9	NP	
LTFLARSAL	9	NP	
LVWMACHSA	9	NP	44.44%–44.44%
MIDGIGRFY	9	NP	
MSFQGRGVF	9	NP	
MSNEGSYFF	9	NP	
MVMELIRMI	9	NP	55.56%–55.56%
NAYERMCNT	9	NP	
NLNDATYQR	9	NP	44.44%–44.44%
NLPFDRTTI	9	NP	
NPIVPSFDM	9	NP	
PAHKSQLVW	9	NP	
QLSTRGVQI	9	NP	33.33%–33.33%
RASAGQISV	9	NP	
RGINDRNFW	9	NP	33.33%–33.33%
RLIQNSITI	9	NP	44.44%–44.44%
RLIQNSLTI	9	NP	44.44%–44.44%
RLLQNSQVY	9	NP	
SARPEDVSF	9	NP	
SPIVPSFDM	9	NP	44.44%–44.44%
SRYWAIRTR	9	NP	33.33%–44.44%
STLELRSGY	9	NP	
STLELRSRY	9	NP	
TFLARSALI	9	NP	55.56%–55.56%
TRALVRTGM	9	NP	
TTYQRTRAL	9	NP	44.44%–44.44%
VLRGSVAHK	9	NP	66.67%–66.67%
VLSFIKGTK	9	NP	
VLSTEKSPF	9	NP	
VSFQGRGVF	9	NP	
WMACHSAAF	9	NP	
WMACNSAAF	9	NP	
YSLVGIDPF	9	NP	
AEIEDLIFLA	10	NP	70.00%–70.00%
AFDERRNKYL	10	NP	
AFEDLRVSSF	10	NP	
ATAGLTHMMI	10	NP	
ATEIRASVGK	10	NP	
DTTYQRTRAL	10	NP	
GQISIQPTFS	10	NP	60.00%–60.00%
LELRSRYWAI	10	NP	30.00%–30.00%
LILRGSVAHK	10	NP	
LPACVYGLAV	10	NP	
LPACVYGPAV	10	NP	
LPRRSGAAGA	10	NP	70.00%–70.00%
MVMELVRMIK	10	NP	
RFSSFIRGKK	10	NP	
RLLSFIRGTK	10	NP	
RMCNILKGKF	10	NP	
RMVLSAFDER	10	NP	40.00%–40.00%
RVLSFIKGTK	10	NP	60.00%–60.00%
RVSSFIRGKK	10	NP	40.00%–40.00%
RVSSFIRGTR	10	NP	50.00%–50.00%
SLVGIDPFRL	10	NP	
SVQPTFSVQR	10	NP	50.00%–50.00%
SVQRNLPFDR	10	NP	
SVQRNLPFER	10	NP	40.00%–40.00%
TFSVQRNLPF	10	NP	
YERMCNILKG	10	NP	40.00%–40.00%
ILYDKEEIRRI	11	NP	
PFDKPTIMAAF	11	NP	36.36%–36.36%
PFEKSTIMAAF	11	NP	36.36%–36.36%
PFERATVMAAF	11	NP	36.36%–36.36%
RMIGGIGRFYI	11	NP	
RRSGAAGAAIK	11	NP	81.82%–81.82%
RRSGAAGAAMK	11	NP	72.73%–72.73%
RRSGAAGAAVE	11	NP	63.64%–63.64%
RRSGAAGAAVK	11	NP	72.73%–72.73%
RTEIIRMMESA	11	NP	
SAFDERRNRYL	11	NP	
TIAMELIRMIK	11	NP	45.45%–45.45%
DVTYQRTRALVR	12	NP	58.33%–58.33%
SLIRPNENPAHK	12	NP	
ATKADYTL	8	PA	
KSVFNSLY	8	PA	
MYSDFHFI	8	PA	
RAMAWTVV	8	PA	
SVFNSLYA	8	PA	
AAICTHLEV	9	PA	
AAMDDFQLI	9	PA	
AESRKLLLI	9	PA	77.78%–77.78%
ALLKHRFEI	9	PA	
APIEHIASM	9	PA	
ASRGLWDSF	9	PA	
CAAMDDFQL	9	PA	
CELTDSSWI	9	PA	33.33%–33.33%
CFMYSDFHF	9	PA	
CLINDPWVL	9	PA	
CMKSFFGWK	9	PA	
CMKTFFGWK	9	PA	
DLKIETNKF	9	PA	
EEMATKADY	9	PA	
ELDEIGEDV	9	PA	
EYIMKGVYI	9	PA	
FLLMDALKL	9	PA	
FMYSDFHFI	9	PA	44.44%–44.44%
FNPMIVELA	9	PA	
FNSFLTHAL	9	PA	
FVRQCFNPM	9	PA	
GEETIEERF	9	PA	
GENMAPEKV	9	PA	
GLYEAIEEC	9	PA	
GTFDLGGLY	9	PA	
GTSKIKMKW	9	PA	
HEGEGIPLY	9	PA	
HEKGINPNY	9	PA	
IASMRRNYF	9	PA	
KPKFLPDLY	9	PA	
KTHIHIFSF	9	PA	
KVCRTLLAK	9	PA	
LAKSVFNSL	9	PA	
LLKHRFEII	9	PA	
LLLIVQALR	9	PA	
LYDYKENRF	9	PA	
MKWGMEMRR	9	PA	
NMAPEKVDF	9	PA	
RARIKTRLF	9	PA	
RIKTRLFTI	9	PA	
RRKTNLYGF	9	PA	
SEKTHIHIF	9	PA	
SHEGEGIPL	9	PA	
SLPPNFSSL	9	PA	
SMIEAESSV	9	PA	
SRARIKTRL	9	PA	
SVKEKDMTK	9	PA	55.56%–55.56%
SYINRTGTF	9	PA	
TRREVHIYY	9	PA	
VSHCRATEY	9	PA	
YINTALLNA	9	PA	44.44%–44.44%
YLMAWKQVL	9	PA	
YVRTNGTSK	9	PA	
YYLEKANKI	9	PA	44.44%–44.44%
CFMYSDFHFI	10	PA	
DVVNFVSMEF	10	PA	
KFLPDLYDYK	10	PA	70.00%–70.00%
LNASWFNSFL	10	PA	
LYASPQLEGF	10	PA	50.00%–50.00%
MLLRSAIGQV	10	PA	
RQEMASRGLW	10	PA	
SCLENFRAYV	10	PA	
SSLENFRAYV	10	PA	
QLMWALGENMA	11	PA	36.36%–36.36%
RIMAWTVVNSI	11	PA	
RTMAWTVVNSI	11	PA	54.55%–54.55%
SMRRNYFTAEV	11	PA	
NVMGMIGI	8	PB2	
NVMGMIGV	8	PB2	
PKIYKTYF	8	PB2	
RMKWMMAM	8	PB2	
RYGPALSI	8	PB2	50.00%–50.00%
SFGGFTFK	8	PB2	
TFDTAQII	8	PB2	
TVQIIKLL	8	PB2	
TYSSSMMW	8	PB2	
VAYMLERE	8	PB2	
VLFQNWGI	8	PB2	
VYIEVLHL	8	PB2	
WIIRNWET	8	PB2	
AARNIVRRA	9	PB2	
AYMLERELV	9	PB2	
CLLQSLQQI	9	PB2	
EEFTMVGRR	9	PB2	
ELVRKTRFL	9	PB2	
FPNEVGARI	9	PB2	
FQKDAKVLF	9	PB2	
FQNWGIESI	9	PB2	
FRNQVKIRR	9	PB2	
FSFGGFTFK	9	PB2	33.33%–33.33%
FVNRANQRL	9	PB2	
FVRTLFQQM	9	PB2	
GPALSINEL	9	PB2	
GPVHFRNQV	9	PB2	
GRQEKNPAL	9	PB2	
GRRATAILR	9	PB2	
GTEKLTITY	9	PB2	
GTFDTVQII	9	PB2	
GTFGPVHFR	9	PB2	
GTSGVESAV	9	PB2	
HEGYEEFTM	9	PB2	
HFQKDAKVL	9	PB2	
IIAARNIVR	9	PB2	
ILPDMTPSI	9	PB2	
ILRKATRRL	9	PB2	
ITKEKKEEL	9	PB2	
ITNTVHYPK	9	PB2	
IYKTYFERV	9	PB2	
KAVRGDLNF	9	PB2	
KTTKRLTIL	9	PB2	
KTYFERVER	9	PB2	
KVYKTYFEK	9	PB2	
KYTSGRQEK	9	PB2	
LMDALKLSI	9	PB2	
LTITYSSSM	9	PB2	
LVRGNSPVF	9	PB2	
MAMRYPITA	9	PB2	
MEFEPFQSL	9	PB2	
MKWMMAMKY	9	PB2	
MLERELVRK	9	PB2	
MMWEINGPK	9	PB2	
NEVGARILT	9	PB2	
NPALRMKWM	9	PB2	
NSPVFNYNK	9	PB2	
QMRDVLGTF	9	PB2	
QYSGFVRTL	9	PB2	66.67%–66.67%
RAAVSADPL	9	PB2	
REILTKTTV	9	PB2	
RELVRKTRF	9	PB2	
RLTILGKDA	9	PB2	
RMKWMMAMR	9	PB2	
RMQFSSFTV	9	PB2	
RNEQGQTLW	9	PB2	
RRATAILRK	9	PB2	
RVMVSPLAV	9	PB2	
RYSGFVRTL	9	PB2	
SADPLASLL	9	PB2	
SETQGTEKL	9	PB2	
SFSFGGFTF	9	PB2	
SLENFRAYV	9	PB2	33.33%–33.33%
SPLMVAYML	9	PB2	
SRTREILTK	9	PB2	44.44%–44.44%
TMLYNKMEF	9	PB2	
TTVDHMAII	9	PB2	
TVDHMAIIK	9	PB2	
TVHYPKIYK	9	PB2	
TYFERVERL	9	PB2	
TYQWIIRNW	9	PB2	55.56%–55.56%
VAGGTSSVY	9	PB2	
VERLKHGTF	9	PB2	
VHFRNQVKI	9	PB2	
VSPLAVTWW	9	PB2	
WMMAMKYPI	9	PB2	
WMMAMRYPI	9	PB2	
WSQNPTMLY	9	PB2	
YLEKANKIK	9	PB2	
YPITADKRI	9	PB2	
YPKIYKTYF	9	PB2	
AIRGQYSGFV	10	PB2	
AMKYPITADK	10	PB2	
APPKQSRMQF	10	PB2	
FPNEVGARIL	10	PB2	
GPPCSQRSKF	10	PB2	
GTFDTAQIIK	10	PB2	
HFQKDAKVLF	10	PB2	
HYPKIYKTYF	10	PB2	30.00%–30.00%
HYPKVYKTYF	10	PB2	
KWMMAMRYPI	10	PB2	
LLMDALKLSI	10	PB2	
LYNKMEFEPF	10	PB2	
NTVHYPKIYK	10	PB2	
QYSGFVRTLF	10	PB2	
RLNPMHQLLR	10	PB2	
RMKWMMAMRY	10	PB2	
RYSGFVRTLF	10	PB2	
SFSFGGFTFK	10	PB2	30.00%–30.00%
SINELSNLAK	10	PB2	
SPKGVEESSI	10	PB2	
VLRGFLILGK	10	PB2	70.00%–70.00%
VMVSPLAVTW	10	PB2	
VVVSIDRFLR	10	PB2	
FTFKRTSGSSV	11	PB2	
GTAGVESAVLR	11	PB2	36.36%–36.36%
KTYFEKVERLK	11	PB2	
PVAGGTSSIYI	11	PB2	63.64%–63.64%
QEKNPALRMKW	11	PB2	81.82%–81.82%
RTLFQQMRDVL	11	PB2	
SILTDSQTATK	11	PB2	
STSGVESAVLR	11	PB2	45.45%–45.45%
TTQIIKLLPFA	11	PB2	
SSILTDSQTATK	12	PB2	
ILPQQFIQK	9	Protein PB1-F2 (PB1-F2)	
KETGERQYD	9	PB1-F2	
STEHINIQK	9	PB1-F2	
STGHISTQK	9	PB1-F2	
TKGAAIYLK	9	PB1-F2	
GCQSDLTFLK	10	PB1-F2	
KILPQQFIQK	10	PB1-F2	
TTLEISTFLK	10	PB1-F2	
TPNFLSPSLEIT	12	PB1-F2	
ALANTIEV	8	PB1	
EFTSFFYR	8	PB1	
FEFTSFFY	8	PB1	87.50%–87.50%
FESKSMKL	8	PB1	50.00%–50.00%
FTSFFYRY	8	PB1	
LASIDLKY	8	PB1	
LLFLKVPA	8	PB1	50.00%–50.00%
MLSTVLGV	8	PB1	
NLYNIRNL	8	PB1	
NPRMFLAM	8	PB1	
NTSQRGIL	8	PB1	
NVLSIAPI	8	PB1	
QYGGGYGF	8	PB1	
QYKGKLCL	8	PB1	
RLGKGYMF	8	PB1	
RYGFVANF	8	PB1	75.00%–75.00%
SFFYRYGF	8	PB1	
STTFPYTG	8	PB1	
TVIKTNMI	8	PB1	75.00%–75.00%
YLIRALTL	8	PB1	
AEIMKICST	9	PB1	33.33%–33.33%
ALTLNTMTK	9	PB1	
AMITYITRK	9	PB1	
APIMFSNKM	9	PB1	
AQNAISTTF	9	PB1	
ARIDARIDF	9	PB1	
ARLGKGYMF	9	PB1	77.78%–77.78%
ATAQMALQL	9	PB1	
ATTHSWIPK	9	PB1	
AVATTHSWI	9	PB1	
CEKLEQSGL	9	PB1	88.89%–88.89%
CYKHNFPLY	9	PB1	
DTVNRTHQY	9	PB1	77.78%–77.78%
DVNPTLLFL	9	PB1	
EEIEITTHF	9	PB1	33.33%–33.33%
EEMGITTHF	9	PB1	
EKFFPSSSY	9	PB1	
EWMSIRPYF	9	PB1	33.33%–33.33%
FIKDYRYTY	9	PB1	
FLAMITYMT	9	PB1	
FLEESHPGI	9	PB1	
FLKDVMESM	9	PB1	33.33%–33.33%
FLKVPAQNA	9	PB1	
FPYTGDPPY	9	PB1	
FVANFSMEL	9	PB1	66.67%–77.78%
FVEALARSI	9	PB1	55.56%–55.56%
FVETLARSI	9	PB1	
FVHFVEALA	9	PB1	
FVYFVETLA	9	PB1	
GITTHFQRK	9	PB1	
GMFNMLSTV	9	PB1	
GMQIRGFVY	9	PB1	
GPATAQMAL	9	PB1	77.78%–77.78%
GTFEFTSFF	9	PB1	
GYMFESKSM	9	PB1	
HIPEVCLKW	9	PB1	
HQYSERGRW	9	PB1	
IPAEMLASI	9	PB1	
IPKRNRSIL	9	PB1	
IQAGVDRFY	9	PB1	
IQYGGGYGF	9	PB1	
ISSLFIDSI	9	PB1	
KDVMESMNK	9	PB1	
KEKDMTKEF	9	PB1	
KFFPSSSYR	9	PB1	
KICSTIEEL	9	PB1	
KLTQGRQTY	9	PB1	
KMARLGKGY	9	PB1	77.78%–77.78%
KTTYWWDGL	9	PB1	
KYTKTIYWW	9	PB1	
LEESHPGIF	9	PB1	
LPSFGVSGI	9	PB1	66.67%–77.78%
MEYDAVATT	9	PB1	
MLASIDLKY	9	PB1	
MMMGMFNML	9	PB1	100.00%–100.00%
MQIRGFVYF	9	PB1	
MYQKCCNLF	9	PB1	
NESGRLIDF	9	PB1	
NLFEKFFPS	9	PB1	
NMLSTVLGV	9	PB1	100.00%–100.00%
NPRMFLAMI	9	PB1	
NTMTKDAER	9	PB1	
QLALQLFIK	9	PB1	
QLFIKDYRY	9	PB1	
QPEWFRNIL	9	PB1	
QPEWFRNVL	9	PB1	44.44%–44.44%
QTYDWTLNR	9	PB1	
RGKLKRRAI	9	PB1	
RLIDFLKDV	9	PB1	
RLNKRSYLI	9	PB1	
RMFLAMITY	9	PB1	
RNQPAATAL	9	PB1	
RPLLIEGTA	9	PB1	
RPVGISSMV	9	PB1	
RRAIATPGM	9	PB1	77.78%–77.78%
RRVRDNMTK	9	PB1	
RVRDNMTKK	9	PB1	
RYTKTTYWW	9	PB1	33.33%–33.33%
SMELPSFGV	9	PB1	
SPGMMMGMF	9	PB1	
SSDDFALIV	9	PB1	
SYLIRALTL	9	PB1	
TFEFTSFFY	9	PB1	77.78%–77.78%
TLARSICEK	9	PB1	55.56%–55.56%
TLNRNQPAA	9	PB1	
TLNTMTKDA	9	PB1	
TQGRQTYDW	9	PB1	
TQIQTRRSF	9	PB1	44.44%–44.44%
TTHSWIPKR	9	PB1	
VNNAVVMPA	9	PB1	
VPAQNAIST	9	PB1	
VSDGGPNLY	9	PB1	77.78%–77.78%
VVQQTRVDK	9	PB1	
WIPKRNRSI	9	PB1	
YMFESKRMK	9	PB1	
YMFESKSMK	9	PB1	
YRYGFVANF	9	PB1	
YRYTYRCHR	9	PB1	
YSHGTGTGY	9	PB1	100.00%–100.00%
ETMEVVQQTR	10	PB1	30.00%–30.00%
EVVQQTRVDK	10	PB1	
EVVQQTRVDR	10	PB1	
FLEESHPGIF	10	PB1	
FNMLSTVLGV	10	PB1	100.00%–100.00%
FPSSSYRRPV	10	PB1	
FSMELPSFGV	10	PB1	80.00%–90.00%
FYRYGFVANF	10	PB1	80.00%–80.00%
GLPVGGNEKK	10	PB1	
GMFNMLSTVL	10	PB1	
GMMMGMFNML	10	PB1	
GTFEFTSFFY	10	PB1	80.00%–80.00%
KFFPSSSYRR	10	PB1	
KLVGINMSKK	10	PB1	90.00%–90.00%
LVSDGGPNLY	10	PB1	80.00%–80.00%
MLSTVLGVSI	10	PB1	
MMMGMFNMLS	10	PB1	
MPAHGPAKNM	10	PB1	
MQIRGFVYFV	10	PB1	
NLFEKFFPSS	10	PB1	
NLYNIRNLHI	10	PB1	
SGLPVGGNEK	10	PB1	
STIEELRRQK	10	PB1	
TSFFYRYGFV	10	PB1	
VNPTLLFLKV	10	PB1	
LSIAPIMFSNK	11	PB1	
RIDFESGRIKK	11	PB1	
RVWGGPENRNK	11	PB1	
SILNLGQKRYTK	12	PB1	

FLUAV-derived peptides published on the IEDB [44] as having positive ‘T cell’ (immunogenic) or ‘MHC-ligand’ (HLA-I binding) assays were collated. Peptide length (number of amino acids [aa]) and the protein each peptide was derived from were also recorded. Immunogenic sequences are shown with purple shading, peptides with modified residues were excluded. Range of minimum and maximum sequence identity of epitopes in 3–5 FLUBV sequences, are shaded based on maximum as follows: 0%–40% (red); 41%–50% (pink); 51%–60% (peach); 61%–70% (orange); 71%–80% (yellow); 81%–90% (violet); 91%–100% (blue). Sequences of FLUBV vaccine strains listed by GISAID [55], 2022–2023 (Northern Hemisphere) to 2026–2027 (Northern Hemisphere), were downloaded and IEDB Conservancy analysis tool [60] was used to determine the sequence identity with FLUAV immunogenic peptides.

**Figure 1:**
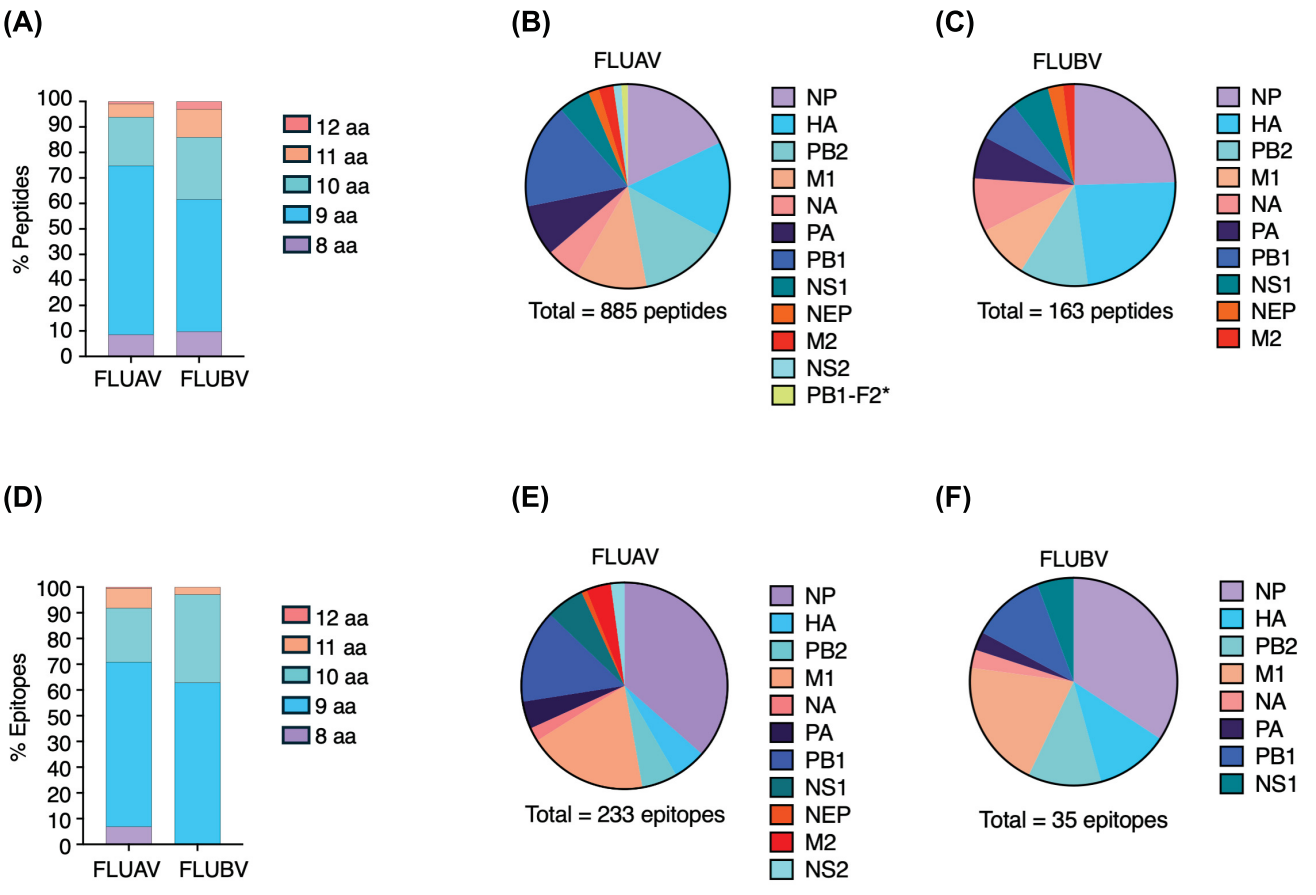
Comparison of length and source protein of peptides and epitopes derived from FLUAV and FLUBV FLUAV- and FLUBV-derived HLA-I-restricted peptide sequences positive for both HLA-Ligand and T cell activation assays (peptides), and only T cell activation assays (epitopes) were downloaded from the IEDB [44]. Peptides and epitopes with modified residues were excluded. All 8–10-mers were included in the analysis. 11–12-mers without overlapping minimal peptides/epitopes were also included. (A–F) The proportion of aa length of peptides (**A**) and epitopes (**D**) derived from FLUAV (*n* = 885 and *n* = 233, respectively) or FLUBV (*n* = 164 and *n* = 35, respectively) was compared. The percentage of FLUAV- (*n* = 885, **B**) or FLUBV-derived (*n* = 163, **C)** peptides or FLUAV- (*n* = 233, **E**) or FLUBV-derived (*n* = 35, **F**) epitopes, from their protein of origin, where reported in the IEDB.

This resulted in 164 (of which 163 had reported protein of origin) and 885 peptides derived from FLUBV and FLUAV, respectively (Tables 1 and 2 and Figure 1A–C). Among those, a respective 21.3% and 26.3% were epitopes from FLUBV (*n* = 35) and FLUAV (*n* = 233) (Tables 1 and 2, Figure 1D–F). Over 97% of the FLUBV-derived epitopes were 9- and 10-mers (Figure 1D), compared with 84.9% for FLUAV-derived epitopes (Figure 1D). Comparatively, 11-mer epitopes were over two-fold more frequent from FLUAV (7.7%) than from FLUBV (2.9%), and while there were 8-mer and 12-mer FLUAV-derived epitopes, no FLUBV-derived epitopes with these lengths have been reported in the IEDB (Table 1 and Figure 1D). Despite these differences, data are consistent with the well-established preference of HLA-I molecules to present peptides of ∼9 residues [45]. This suggests that the absence of FLUBV-derived 8-mer epitopes is likely due to the smaller number of characterised FLUBV epitopes compared with FLUAV. Of the 35 FLUBV-derived CD8^+^ T cell epitopes, 24 have HLA-I restrictions reported in the IEDB [44], represented across five different HLA-I molecules, namely HLA-A*02:01, -A*03:01, -A*11:01, -A*24:02, and -B*15:01, with a cumulative coverage of 53.5% of the global population (Table 1).

FLUAV and FLUBV, while genetically distinct from one another, have several proteins that are functionally homologous [46]. As such, while significantly fewer FLUBV peptides and epitopes have been identified, it is striking that the influenza proteins from which the majority were derived were consistent across both FLUBV and FLUAV (Figure 1B,C,E,F).

NP was the most common source of all peptides, representing 24.5% and 18.0% of all peptides derived from FLUBV and FLUAV, respectively (Figure 1B,C). Similarly, NP-derived epitopes were the most common FLUBV- and FLUAV-derived epitopes, representing 34.3% and 36.5% of epitopes, respectively (Figure 1E,F). Interestingly, M1-derived epitopes were the second most common FLUBV and FLUAV-derived epitopes, representing 20.0% and 18.9%, respectively (Figure 1E,F), despite representing only 8.6% and 11.3% of the total peptides, respectively (Figure 1B,C). Similarly, PB1-derived peptides accounted for only 6.7% of all FLUBV peptides and ranked seventh (Figure 1C) but represented 11.4% of FLUBV-derived epitopes (ranked third, Figure 1F). Conversely, for FLUAV, PB1 was the second most common source of peptides and the third most common source of epitopes (Figure 1B–E). It is interesting that despite the difference in numbers of FLUAV versus FLUBV-derived peptides and epitopes, NP, M1, and PB1 proteins are consistently well represented (Figure 1E,F). Although more work needs to be done, this suggests that these proteins might represent the best source of CD8^+^ T cell epitopes across influenza strains and could be useful for vaccine and therapeutic developments.

## Structures of FLUBV-derived peptides presented by HLA-I molecules

Structural analysis of peptide–HLA complexes can provide a molecular understanding of factors that may influence immunogenicity, such as the overall stability of the peptide–HLA complex, characteristics of the peptide residues available for TCR interactions, and understanding the impact of viral mutations on the immune response. To assess these features of FLUBV-derived peptides, we compiled a list of the known crystal structures of FLUBV-derived peptides presented by HLA-I molecules from the Protein Data Bank (PDB) (Table 3) [47]. Currently, there are only 11 crystal structures of FLUBV-derived peptides in complex with an HLA-I, and none in complex with a TCR (Table 3). This is in sharp contrast with FLUAV-derived peptides, for which there are >50 structures in complex with HLA-I, across 17 HLA-I, including HLA-A, HLA-B, and HLA-C allomorphs.

**Table 3:** Structures available of FLUBV-derived peptides presented by HLA-I molecules

FLUBV-derived peptides					FLUAV-derived peptides					
Peptide	Sequence	HLA-	PDB code	*T*m (°C)	Peptide	Sequence	HLA-	PDB code	*T*m (°C)	Ref
B/PB2_550–558_	TYQWVLKNL	A*24:02	6XQA	57.1 ± 1.2	A/PB2_549–557_	TYQWIIRNW	A*24:02	7JYW	61.9 ± 1.3	[49]
B/NP_164–173_	IYFSPIRVTF	A*24:02	7JYU	64.0 ± 2.2	NA					[49]
B/NP_165–173_	YFSPIRVTF	A*24:02	7JYV	57.5 ± 0.3	NA					[49]
B/NP_511–520_	KTNGNAFIGK	A*11:01	7S8Q	61.3 ± 0.2	NA					[50]
B/M1_41–49_	SALEWIKNK	A*11:01	7S8R	57.6 ± 0.1	NA					[50]
B/NS1_186–195_	RVLVNGTFLK	A*11:01	7S8S	61.3 ± 0.5	NA					[50]
B/NP_323–331_	VVRPSVASK	A*03:01	7MLE	53.3 ± 0.3	A/NP_265–273_	ILRGSVAHK	A*03:01	7UC5	53.9 ± 0.1	[53]
B/NS1_196–206_	HPNGYKSLSTL	B*07:02	8TUB	NA	NA					[51]
B/NP_30–38_	RPIIRPATL	B*07:02	8TUH	NA	NA					[51]
B/M1_58–66_	ALIGASICF	B*15:01	7XF3	NA	A/M1_58–66_	GILGFVFTL	A*02:01	1HHI	65.8 ± 1.2	[54]
B/PB1_177–185_	PEMTFFSVK^#^	B*18:01	9DY8	48.9 ± 1.2	A/PB1_177–185_	EEIEITTHF	B*18:01	8RNH	64.5 ± 0.9	[55]

FLUBV-derived peptides in complex with HLA-I molecules as per the PDB. *T*m, thermal midpoint temperature. NA, not available. #Only the B/PB1_177–185_ was reported to not be immunogenic, the other FLUBV epitopes were immunogenic.

**Figure 2:**
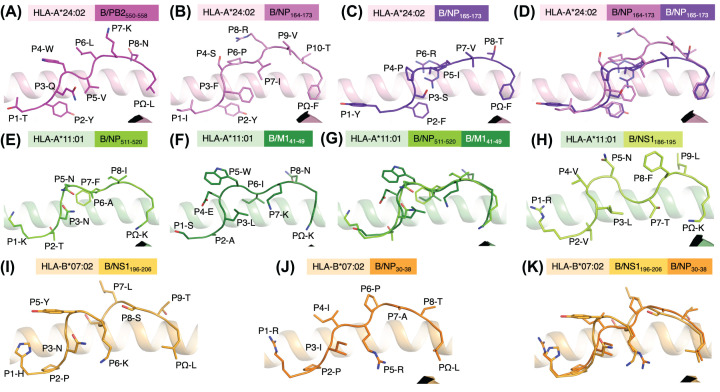
FLUBV-derived peptides in complex with HLA-A*24:02, HLA-A*11:01, and HLA-B*07:02 molecules Structures of FLUBV epitopes in complex with HLA-I molecules were obtained from the PDB [47]. HLA-A*24:02 structure (pale cartoon) in complex with B/PB2_550–558_ epitope (magenta) (**A**), B/NP_164–173_ epitope (pink) (**B**) and B/NP_165–173_ epitope (violet) (**C**). (**D**) Overlay of the HLA-A*24:02-B/NP_164–173_ and HLA-A*24:02-B/NP_165–173_ structures. HLA-A*11:01 (pale green cartoon) in complex with B/NP_511–520_ (chartreuse) (**E**) and B/M1_41–49_ (forest) (**F**) epitopes, respectively, and an overlay of the two complexes (**G**). (**H**) HLA-A*11:01 in complex with B/NS1_186–195_ epitope (light green). HLA-B*07:02 (pale orange cartoon) in complex with the B/NS1_196–206_ (light orange) (**I**) and B/NP_30–38_ epitope (dark orange) (**J**). (**K**) Overlay of the HLA-B*07:02-B/NS1_196–206_ and HLA-B*07:02-B/NS1_30–38_ structures.

HLA-A*24:02 has been solved in complex with three FLUBV-derived peptides (Table 3), two derived from NP, and one from the PB2 protein. The three peptides all shared the favoured anchor residue at P2 (aromatic) and PΩ (hydrophobic) [48] and bound in a canonical fashion to HLA-A*24:02 (Figure 2A–C). The FLUBV PB2_550-558_ (B/PB2_550-558_, TYQWVLKNL) peptide binds stably to the HLA-A*24:02 with a thermal stability of 57°C. The central P5-Val acted as a secondary anchor residue, while the residues P4-Trp, P6-Leu, P7-Lys, and P8-Asn were solvent exposed and therefore available for TCR interaction (Figure 2A). The structures of two overlapping NP-derived peptides in complex with HLA-A*24:02 are also available, namely the 9-mer NP_165–173_ (B/NP_165–173_, YFSPIRVTF) and the 10-mer NP_164–173_ (B/NP_164–173_, IYFSPIRVTF) that has an extra N-terminal residue compared with NP_165–173_ (Table 3 and Figure 2B,C). The structure reveals that the additional N-terminal residue in the 10-mer B/NP_164–173_ drives a distinct conformation of the peptide, with the largest difference observed for the peptide central region (P6–P7) exhibiting a maximum displacement of 3.9 Å for the Cα of the central arginine (Figure 2D). The difference of structure was associated with a less stable conformation formed with the HLA-A*24:02 by the B/NP_165–173_ compared with B/NP_164–173_, with a *T*m difference of 7°C, due to a small P3-Ser secondary anchor compared with a more favourable larger P3-Phe, respectively (Figure 2B,C). While both peptides were immunogenic, their different conformation and stability suggest that they would be recognised by different TCRs [49].

Three HLA-A*11:01-restricted FLUBV peptide structures were also determined, with the B/NP_511–520_, B/M1_41–49_, and B/NS1_186–195_ (Table 3 and Figure 2E–H). Each peptide was able to activate CD8^+^ T cells; however, the responses were most commonly single-function [50]. B/M1_40–49_ stimulated a slightly higher proportion of CD8^+^ T cell responses than B/NP_511–520_ or B/NS1_186–195_ [50]. The three peptides exhibited a relatively high *T*m (57°C–61°C) and therefore stable binding within the HLA-A*11:01 cleft (Table 3), in agreement with the HLA favoured P2 hydrophobic residue and PΩ positively charged residue [48]. Interestingly, despite being of different length, the B/NP_511–520_ (10-mer) and the B/M1_40–49_ (9-mer), the peptide backbones were similar, adopting a flat conformation in the cleft (Figure 2E–G). On the other hand, the B/NS1_186–195_ peptide adopted a distinct conformation to NP- and M1-derived peptides, with the P7-Thr side chain binding deep into the cleft acting as a secondary anchor residue, and the P4-Val, P5-Asn, P8-Phe, and P9-Leu residues were all solvent exposed (Figure 2H).

There are two FLUBV-derived peptides in complex with HLA-B*07:02 for which the crystal structures have been solved, the 9-mer NP_30–38_ (B/NP_30–38_, RPIIRPATL) and the 11-mer NS1_196–206_ (B/NS1_196–206_, HPNGYKSLSTL) (Table 3 and Figure 2I,J) [51]. Both B/NP_30–38_ and B/NS1_196–206_ were immunogenic, activating T cell responses and were characterised by a high magnitude of tetramer^+^ CD8^+^ T cells in T cell lines expanded using peptide pools [51]. Further, a high proportion of *ex vivo* enriched tetramer^+^ CD8^+^ T cells showed a central memory phenotype [51]. The B/NP_30–38_ was shown to be also restricted by HLA-B*08:01 [51,52], but unfortunately, the structure of this complex is not available for comparison with HLA-B*07:02. The structures of HLA-B*07:02 in complex with the B/NP_30–38_ and B/NS1_196–206_ epitopes show that both peptides bind HLA-B*07:02 with canonical primary anchor residues (P2-Pro and PΩ-Leu) in the same conformation (Figure 2K) [48]. Despite the length differences of the two epitopes (9- and 11-mer), they both used a large positively charged (lysine or arginine) residue as secondary anchor (Figure 2I–K), which would stabilise the peptide via the formation of a salt bridge with the HLA Asp114.

**Figure 3:**
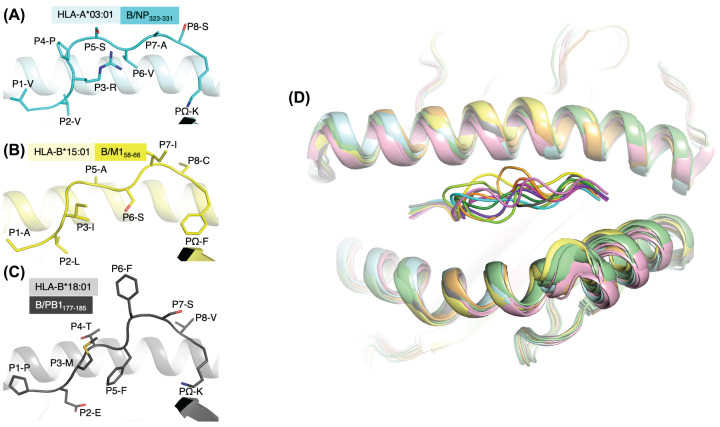
FLUBV-derived peptides in complex with HLA-A*03:01, HLA-B*15:01, and HLA-B*18:01 molecules Structures of FLUBV peptide and epitopes in complex with HLA-I molecules were obtained from the PDB [47]. (**A**) HLA-A*03:01 (pale cyan cartoon) bound to B/NP_323–331_ epitope (cyan). (**B)** HLA-B*15:01 (pale yellow cartoon) in complex with B/M_58–66_ epitope (yellow). (**C**) HLA-B*18:01 (pale grey cartoon) in complex with B/PB1_177–185_ peptide (dark grey). (**D**) Overlay of all FLUBV-derived peptide–HLA complexes in the PDB [47].

Three additional structures of FLUBV-derived peptides in complex with HLA-A*03:01, HLA-B*15:01 and HLA-B*18:01 are available (Table 3 and Figure 3A–C). The three different HLAs present peptides from different FLUBV proteins, namely NP, M1 and PB1, respectively. HLA-A*03:01-B/NP_323–331_ structure showed that the B/NP_323–331_ peptide (VVRPSVASK) contained the canonical hydrophobic P2 residue and a PΩ-Lys characteristic of HLA-A*03:01-restricted peptides (Figure 3A) [48]. The peptide was flat in the HLA cleft, with minimal side chain exposure to the solvent with small hydrophobic residues such as P4-Pro, P5-Ser, P7-Ala and P8-Ser available for TCR recognition (Figure 3A). This featureless conformation could be the basis for the low level of T cell activation toward the B/NP_323–331_ peptide [53].

The HLA-B*15:01 structure has been solved in complex with the FLUBV-derived M1_58–66_ (B/M1_58–66_, ALIGASICF; Table 3 and Figure 3B) and has one of the favoured P2 (P2-QMLVI) and PΩ residues (PΩ-YFM) preferred by the peptides restricted to the HLA-B*15:01 molecule [48]. The B/M1_58–66_ peptide was found to be immunodominant in HLA-B*15:01^+^ samples [54]. The HLA-B*15:01-B/M1_58–66_ structure showed that the peptide uses the central P6-Ser as a secondary anchor to bind to the HLA cleft and that the residues exposed to the solvent were small or hydrophobic, revealing a featureless surface for the TCR to engage with (Figure 3B). The HLA-B*18:01 structure has been solved in complex with the B/PB1_177–185_ peptide (PEMTFFSVK, Figure 3C) that has the P2-Glu favoured by HLA-B*18:01 but has a PΩ-Lys that is unusual for this HLA, which would prefer a large tyrosine, phenylalanine, or methionine residue [48]. The HLA-B*18:01-B/PB1_177–185_ had low thermal stability (*T*m of ∼49°C, Table 3) compared with other HLA-B*18:01-restricted peptides that contained the favoured P2 and PΩ residues [55]. Interestingly, while the peptide conformation allows for the large P6-Phe to be accessible for TCR contact, as well as P4-Thr and P7-Ser that could both form hydrogen bonds with a TCR, the B/PB1_177–185_ peptide was not immunogenic in the samples tested [55].

Overall, the FLUBV-derived peptides adopted a canonical extended conformation within the HLA antigen-binding cleft, in which the peptides are anchored by residues at P2 and PΩ in the cleft and its backbone lies relatively flat while still displaying diversity of conformations (Figure 3D).

## Comparison of FLUBV- and FLUAV-derived homologous peptides

The similarities between FLUAV and FLUBV highlight the potential to leverage the existing work on FLUAV to inform FLUBV CD8^+^ T cell epitope identification, and this strategy has been the basis of many studies looking at the immune response to FLUBV [49–51,53–55]. Of the 11 FLUBV-derived peptides for which a peptide–HLA-I complex structure is available and described above, 4 of them have homologous FLUAV-derived structures available for comparison.

**Figure 4:**
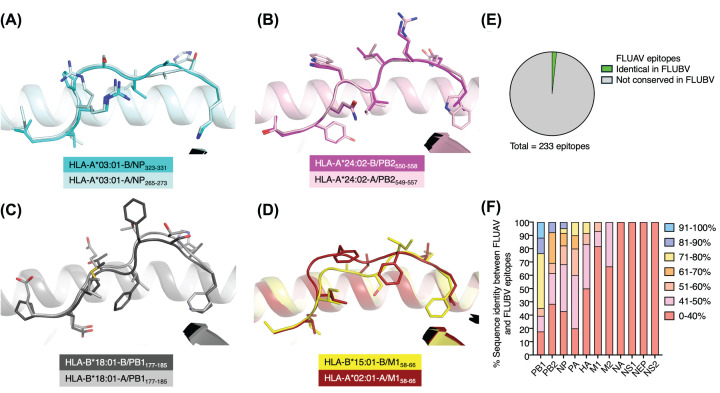
Structures and sequences comparison of FLUAV and FLUBV homologous peptides (**A-D**) Structures of FLUBV peptides in complex with HLA-I molecules were obtained from the PDB [47]. (**A**) Overlay of HLA-A*03:01 in complexes with the FLUBV-derived epitope B/NP_323–331_ (cyan) and FLUAV-derived epitope A/NP_265–273_ (light cyan). (**B**) Overlay of HLA-A*24:02 bound to FLUBV epitope B/PB2_550–558_ (magenta) and FLUAV epitope PB2_549–557_ (light pink). (**C**) Overlay of HLA-B*18:01 presenting a FLUBV peptide B/PB1_177–185_ (dark grey) and FLUAV epitope A/PB1_177–185_ (light grey). (**D**) Superimposition of HLA-B*15:01 in complex with the FLUBV epitope M_58–66_ (yellow) and FLUAV epitope M_58–66_ (brick). (**E,F**) Sequences of FLUBV 2022–2023 to 2026–2027 vaccine strains obtained through GISAID [59] (B/Tokyo/EIS13-175/2025 (B/Victoria lineage)-like EPI_ISL_20373028, B/Pennsylvania/14/2025 (B/Victoria lineage)-like EPI_ISL_20077099, B/Austria/1359417/2021 (B/Victoria lineage)-like EPI_ISL_1519459, B/Austria/1359417/2021 (B/Victoria lineage)-like EPI_ISL_983345, B/PHUKET/3073/2013 (B/Yamagata lineage)-like EPI_ISL_168822, B/PHUKET/3073/2013 (B/Yamagata lineage)-like EPI_ISL_161843) were downloaded and IEDB Conservancy analysis tool [58] was used to determine the minimum and maximum degree of aa similarity (sequence identity) between the protein sequences of these vaccine strains and the FLUAV epitopes from the corresponding protein of origin. Sequence identity was analysed per protein, and duplicated sequences were removed, leaving 3–5 sequences depending on the protein. (**E**) Percentage of total FLUAV epitopes conserved in FLUBV vaccine strains with 100% sequence identity. (**F**) Proportion of epitopes with different % sequence identity separated per protein.

Firstly, the HLA-A*03:01-restricted B/NP_323–331_ (VVRPSVASK) has a FLUAV homologous epitope, namely the well-characterised A/NP_265–273_ (ILRGSVAHK), sharing 55% sequence identity and 78% similarity (Table 3) [53]. Although both peptides effectively stabilise the HLA-A*03:01 molecule with comparable thermal stability (*T*m ∼ 53°C–54°C, Table 3), the B/NP_323–331_ was shown to be less immunogenic than the A/NP_265–273_ counterpart [53]. Furthermore, the A/NP_265-273_ peptide expanded a greater proportion of tetramer^+^CD8^+^ T cells than the B/NP_323–331_ epitope in T cell lines [53]. Interestingly, a small degree of CD8^+^ T cell cross-reactivity was observed towards the two epitopes, potentially driven by the similar conformation of the peptides within the HLA-A*03:01 binding cleft (Figure 4A). Both peptides exhibit a relatively similar flat conformation with a root mean square deviation (r.m.s.d.) of only 0.37 Å for the peptide. While the A/NP_265–273_ differs from the B/NP_323–331_ peptide by four residues (Table 3), the high degree of conformational similarity likely facilitated a level of CD8^+^ T cell cross-reactivity [53].

The second example includes FLUAV- and FLUBV-derived homologous peptides in complex with HLA-A*24:02. The FLUAV-derived A/PB2_549–557_ (TYQWIIRNW) and FLUBV-derived B/PB2_550–558_ (TYQWVLKNL) epitopes share 55% sequence identity [49]. Both peptides adopted very similar conformations in the HLA-A*24:02 cleft (r.m.s.d. of 0.32 Å for the peptide), but the different C-terminal anchor (PΩ) residues impacted their stability (Figure 4B and Table 3) [49]. Indeed, the A/PB2_549–557_ peptide contained the most favoured PΩ for binding to the F pocket of HLA-A*24:02, namely tryptophan [48], and therefore had a higher *T*m than that observed for the HLA in complex with the B/PB2_550–558_, which contained a leucine at PΩ (Table 3). Despite these anchor residue variations, the similar overall conformation of the two PB2 peptides provides a basis for the T cell cross-reactivity observed [49].

The third example is for the HLA-B*18:01-restricted B/PB1_177–185_ peptide, which was identified as a homologue of a previously characterised HLA-B*18:01-restricted A/PB1_177–185_ epitope [55]. While the B/PB1_177–185_ peptide was identified from the corresponding region of the FLUBV PB1 protein to the FLUAV-derived epitope, the homologous peptides have limited sequence identity, only sharing the anchor residue P2-Glu, and not the PΩ residue (Table 3 and Figure 4C). Consequently, the B/PB1_177–185_ peptide contains a less favoured PΩ-Lys than the PΩ-Phe in the A/PB1_177–185_ epitope, decreasing the overall peptide–HLA complex stability by ∼15°C (Table 3) [55]. In addition to the less favoured PΩ-Lys anchor residue, the presence of P5-Phe acting as a secondary anchor is also unfavoured in the HLA-B*18:01 binding cleft [48], and this, in turn, creates some mobility in the central part of the peptide. This increased flexibility is likely to hinder TCR engagement and could lead to the lack of CD8^+^ T cell activation observed towards B/PB1_177–185_ [55]. Interestingly, despite the high sequence variation between the B/PB1_177–185_ and A/PB1_177–185_ peptides, their backbones adopted a similar structure (Figure 4C).

The last example is of the HLA-B*15:01-restricted B/M1_58–66_ (ALIGASICF) epitope [54], for which the FLUAV-derived homologue is the immunodominant A/M1_58–66_ (GILGFVFTL) epitope that is restricted to and has been solved in complex with HLA-A*02:01 (Table 3 and Figure 4D) [56,57]. Interestingly, while these homologous epitopes were restricted by distinct HLA molecules, their orientation in the binding cleft of each allomorph was similar, as was their ‘flat and featureless’ conformation (Figure 4D). In HLA-B*15:01, the B/M1_58–66_ epitope is anchored by P2-Leu and P9-Phe, while P3-Ile and P6-Ser acted as secondary anchor residues [54]. The B/M1_58–66_ residues at P4, P5, P7, and P8 were solvent exposed, mirroring the A/M1_58–66_ structure [54]. Even if the backbone was differed between the epitopes, both had a flat conformation with limited long side chains exposed to the solvent for TCR binding. Both epitopes were able to activate T cells when presented by their respective HLA allomorphs, but B/M1_58–66_ was not immunogenic in HLA-A*02:01^+^ samples [54], likely due to the different anchor residue requirements between the two HLA molecules.

The crystal structures of FLUBV-derived peptides show an overall canonical peptide binding pattern in the HLA cleft compared with FLUAV-derived peptides. However, conformation and sequence differences influence the level of immunogenicity of FLUBV-derived epitopes compared with their FLUAV homologues.

From these few examples, it is clear that homologous epitopes are not always conserved and that even minimal amino acid changes can affect the structure of an epitope within its respective HLA-I and its ability to stimulate CD8^+^ T cells. To look at whether other FLUAV-derived epitopes that do not have structures are conserved in FLUBV, we used the IEDB epitope conservancy analysis tool [58] to compare their sequences with FLUBV vaccine strains obtained through GISAID [59]. We found that less than 2% of FLUAV epitopes are identical in the FLUBV vaccine strains (Table 2 and Figure 4E), and only ∼15% of FLUAV epitopes had high (≥70%) sequence identity in FLUBV (Figure 4F). Of the FLUAV-derived epitopes, the ones from PB1 were most conserved in FLUBV, with ∼11% of the epitopes with complete sequence identity and ∼60% of the epitopes having ≥50% sequence identity (Figure 4F).

Overall, this highlights that in order to understand and protect against FLUBV we cannot simply rely on knowledge from FLUAV, and there is a great need to identify and study FLUBV epitopes in their own right.

## Conclusion

Many studies have thus far focused on the identification and characterisation FLUAV-derived epitopes; however, the role of CD8^+^ T cells in the response to FLUBV is comparatively understudied [49–55,60,61]. In humans, several studies have directly implicated CD8^+^ T cells in the reduction of FLUAV disease severity [32,35,36]. The magnitude and frequency of epitope-specific CD8^+^ T cells have been inversely correlated with clinical symptoms in both seasonal (H1N1) and avian (H7N9) strains of FLUAV [35,36]. Conversely, during an H7N9 outbreak, the absence of epitope-specific CD8^+^ T cells was associated with increased morbidity and mortality [36]. Furthermore, a recent study tracking cellular correlates of protection throughout an influenza season in New Zealand found that CD8^+^ T cells correlated with protection against symptomatic influenza in both the unvaccinated and vaccinated groups [32]. In particular, polyfunctional CD8^+^ T cells were associated with protection, while single-function CD8^+^ T cells were linked to disease susceptibility, demonstrating the importance of the quality of T cell responses in viral control [32].

In contrast, despite FLUBV being responsible for ∼25% of seasonal influenza infections, and in some years, up to 80% of seasonal infections [15,19], there is limited understanding of the T cell-driven response towards FLUBV, particularly in human cohorts. In mice, CD8^+^ T cells are required for the control of FLUBV and have also been implicated in reducing viral shedding, accelerating viral clearance, and reducing cytokine storms involved in influenza-related immunopathology during FLUBV challenge [62,63].

The first step to fill this gap in knowledge is to identify CD8^+^ T cell epitopes derived from FLUBV. According to the IEDB [44], over 233 epitopes have been characterised for FLUAV, while only 35 FLUBV-derived epitopes are known to date, and there are only 11 FLUBV-derived structures in the PDB [47]. Further, many of the FLUBV epitopes have been identified by searching FLUAV epitope homologues rather than through bona fide epitope discovery. This strategy has merit and has led to the discovery of some epitopes; however, these FLUBV-derived epitopes were generally less immunogenic than their FLUAV counterparts [53,55]. This highlights that more work is required to identify potent FLUBV epitopes that could potentially provide a robust protective CD8^+^ T cell response.

The second step in addressing this knowledge gap requires detailed characterisation of FLUBV peptides to provide a framework for antigen presentation, peptide flexibility, and immunogenicity. From the emerging information available, we found that despite the differences between FLUAV and FLUAB, the known epitopes are mainly derived from the same proteins, namely NP, M1, and PB1. This offers a potential to focus on these specific proteins in search of immunodominant FLUBV-derived epitopes. Comparing the 11 FLUBV-derived peptide–HLA-I structures, we can see that the FLUBV-derived peptides bind canonically into the HLA-I cleft via primary anchor residues at P2 and PΩ. Some of the FLUBV-derived peptides were able to stabilise the HLA-I molecule to the same level as their FLUAV counterparts, and to activate CD8^+^ T cells, while the B/PB1_177–185_ peptide exhibiting the lowest *T*m among the FLUBV-derived peptides was not immunogenic in the samples tested [55]. In the limited number of crystal structures and biophysical data available so far for FLUBV-derived peptides, it appears that they observe the same ‘rules’ as FLUAV-derived peptides in the way they interact with the HLA-I molecules. Interestingly, the M1_58–66_ peptides from FLUAV and FLUBV were not restricted to the same HLA-I, namely HLA-A*02:01 and HLA-B*15:01, respectively. The peptide sequences are conserved at only a single residue, and although the peptide structures were distinct, they were both overall featureless and able to strongly activate CD8^+^ T cells [54]. This shows that, in the search for FLUBV-derived peptides that are FLUAV homologues, one should also consider the HLA-I restriction, which may change depending on the level of sequence homology. Therefore, while some FLUBV-derived peptides might not be immunogenic in the samples tested, they might be in the context of a different HLA-I molecule. There is more research needed to understand if this is a general phenomenon or an isolated example. In addition, there is no structure of a TCR engaging any FLUBV-derived peptides in complex with HLA-I. This represents a critical gap in understanding the molecular mechanism of FLUBV-specific CD8^+^ T cell immunity.

Overall, data on FLUBV-derived peptides are emerging and show some similarity to their FLUAV counterparts, but they also indicate that studies focusing on identifying bona fide FLUBV epitopes will be required to extend the characterisation of homologous peptides from FLUAV.

## Perspectives

- Importance of the field: Despite both FLUAV and FLUBV contributing to the significant morbidity and mortality of influenza disease, FLUBV is significantly understudied. It is important to understand both FLUAV and FLUBV to inform future vaccines and therapeutics against influenza viruses.
- Current thinking: FLUAV-derived epitopes have been the focus of many studies.
- Future directions: There is a need to identify and characterise bona fide FLUBV-derived epitopes at the functional and molecular levels.

## Open Access

Open access for this article was enabled by the participation of La Trobe University in an all-inclusive Read & Publish agreement with Portland Press and the Biochemical Society under an agreement with CAUL.

## Abbreviations

HA: haemagglutinin
HLA-I: human leukocyte antigen class I
IEDB: Immune Epitope Database
IFNγ: interferon-γ
IL-2: interleukin-2
M1: Matrix protein 1
M2: Matrix protein 2
NA: neuraminidase
NEP: nuclear export protein
NS1: non-structural protein 1
NP: nucleoprotein
P2: position 2
PA: polymerase acidic protein
PDB: Protein Data Bank
PB1: RNA-directed RNA polymerase catalytic subunit
PB1-F2: protein PB1-F2
PB2: polymerase basic protein 2
r.m.s.d.: root mean square deviation
TCR: T cell receptor
TNF: tumour necrosis factor
FLUAV: *Alphainfluenzavirus influenzae*
FLUBV: *Betainfluenzavirus influenzae*
FLUCV: *Gammainfluenzavirus influenzae*
FLUDV: *Deltainfluenzavirus influenzae*
